# Supplementary material for: The interactome of intact mitochondria by cross-linking mass spectrometry provides evidence for coexisting respiratory supercomplexes
Source: Mol Cell Proteomics. 2017 Dec 8;17(2):216–32. doi: 10.1074/mcp.RA117.000470 (PMC5795388; doi:10.1074/mcp.RA117.000470)
Supplement: Supplemental Data [file supp_RA117.000470_133922_0_supp_23978_fzffwf.zip › spectra_annotation/mito_DR_spectra_annotation/221-1-4-1-5-1.pdf]

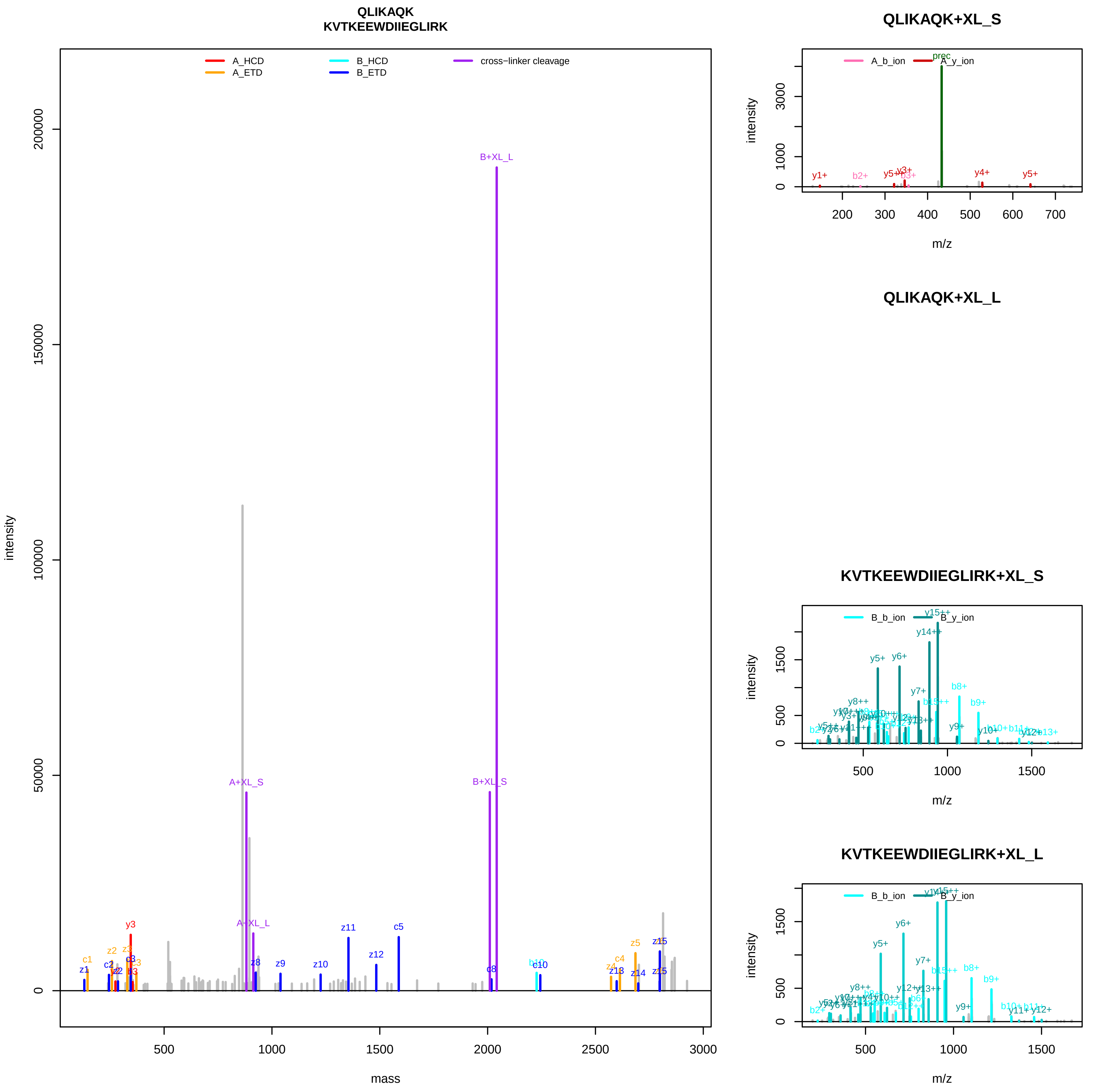
QLIKAQK
KVTKEEWDIIEGLIRK
A_HCD
A_ETD
B_HCD
B_ETD
cross−linker cleavage
intensity
mass
0
50000
100000
150000
200000
500
1000
1500
2000
2500
3000
B+XL_L
A+XL_S
B+XL_S
A+XL_L
y3
c1
z1
c2
z2
z3
c3
z8
z9
z10
z11
z12
c5
c8
c10
z4
c4
z5
z13
z14
z15
QLIKAQK+XL_S
A_b_ion
A_y_ion
prec
y1+
b2+
y5++
y3+
b3+
y4+
y5+
m/z
0
1000
3000
200
300
400
500
600
700
QLIKAQK+XL_L
KVTKEEWDIIEGLIRK+XL_S
B_b_ion
B_y_ion
y15++
y14++
y5+
y6+
y7+
y8++
b8+
b9+
b15++
y9+
y10+
b11+
y12+
b13+
1500
1000
500
KVTKEEWDIIEGLIRK+XL_L
y12++
y13++
b10+
y11+
